# Potential Associations between Severity of Infection and the Presence of Virulence-Associated Genes in Clinical Strains of *Staphylococcus aureus*

**DOI:** 10.1371/journal.pone.0018673

**Published:** 2011-04-26

**Authors:** Steven R. Gill, Lauren M. McIntyre, Charlotte L. Nelson, Brian Remortel, Tom Rude, L. Barth Reller, Vance G. Fowler

**Affiliations:** 1 Department of Oral Biology, University at Buffalo, Buffalo, New York, United States of America; 2 Department of Microbiology and Immunology, University at Buffalo, Buffalo, New York, United States of America; 3 NYS Center of Excellence in Bioinformatics and Life Sciences, University at Buffalo, Buffalo, New York, United States of America; 4 Molecular Genetics and Microbiology, University of Florida, Gainesville, Florida, United States of America; 5 Duke Clinical Research Institute, Duke University Medical Center, Durham, North Carolina, United States of America; 6 J. Craig Venter Institute, Rockville, Maryland, United States of America; 7 Division of Infectious Diseases, Department of Medicine, Duke University Medical Center, Durham, North Carolina, United States of America; 8 Clinical Microbiology Laboratory, Duke University Medical Center, Durham, North Carolina, United States of America; University of Liverpool, United Kingdom

## Abstract

**Background:**

The clinical spectrum of *Staphylococcus aureus* infection ranges from asymptomatic nasal carriage to osteomyelitis, infective endocarditis (IE) and death. In this study, we evaluate potential association between the presence of specific genes in a collection of prospectively characterized *S. aureus* clinical isolates and clinical outcome.

**Methodology/Principal Findings:**

Two hundred thirty-nine *S. aureus* isolates (121 methicillin-resistant *S. aureus* [MRSA] and 118 methicillin-susceptible *S. aureus* [MSSA]) were screened by array comparative genomic hybridization (aCGH) to identify genes implicated in complicated infections. After adjustment for multiple tests, 226 genes were significantly associated with severity of infection. Of these 226 genes, 185 were not in the SCC*mec* element. Within the 185 non-SCC*mec* genes, 171 were less common and 14 more common in the complicated infection group. Among the 41 genes in the SCC*mec* element, 37 were more common and 4 were less common in the complicated group. A total of 51 of the 2014 sequences evaluated, 14 non-SCC*mec* and 37 SCC*mec*, were identified as genes of interest.

**Conclusions/Significance:**

Of the 171 genes less common in complicated infections, 152 are of unknown function and may contribute to attenuation of virulence. The 14 non-SCC*mec* genes more common in complicated infections include bacteriophage-encoded genes such as regulatory factors and autolysins with potential roles in tissue adhesion or biofilm formation.

## Introduction

The clinical spectrum of *Staphylococcus aureus* is diverse, ranging from asymptomatic colonization to osteoarticular infections, endocarditis, and death [1], [2], [3], [4], [5], [6]. Although a growing body of evidence suggests that bacterial genetic characteristics may be associated with distinct clinical manifestations of *S. aure*us [7], [8], [9], [10], [11], [12], [13], the association between *S. aureus* genes and severity of illness is incompletely understood.

Using multi-locus sequence typing (MLST), we previously demonstrated a significant association between specific clonal complexes (CC) and severity of infection in *S. aureus* [9]. Among 371 clinically well-characterized *S. aureus* from diverse clinical settings, we showed that CC5 and CC30 were significantly associated with hematogenous complications of *S. aureus* such as endocarditis, septic arthritis, and vertebral osteomyelitis. Isolates within these CCs were also implicated using staphylococcal protein A (*spa*) [14] and staphylococcal cassette chromosome *mec* (SCC*mec*) typing [15]. However, the genetic basis for these associations is unknown.

The current investigation seeks to evaluate potential associations between the presence of specific genes and clinical outcome. To do this, we used a novel array comparative genome hybridization (aCGH) analysis method to identify individual genes in a large collection of clinically well-characterized *S. aureus* isolates from a wide range of infection severity [16]. We hypothesized that the association between CC5 and CC30 and hematogenous complications was due to the presence of specific bacterial genes contained within these clonal backgrounds, and tested this hypothesis for over 2,000 genes in the genomes of 239 clinical *S. aureus* isolates.

## Methods

### Subject characteristics

This study was approved by the Duke University Medical Center (DUMC) Institutional Review Board. Written informed consent was obtained from all participants involved in this study. At Duke Medical Center, hospitalized adult patients with at least one blood culture positive for *S. aureus* were prospectively identified between September 1994 and October 2003 [17]. Bacterial bloodstream isolates were cataloged, stored and corresponding clinical data were prospectively collected [17]. Twelve weeks after initial positive blood culture, clinical outcomes were prospectively assessed. Patients were excluded from the investigation for the following reasons: outpatient status, age <18 years, polymicrobial infection, neutropenia (absolute neutrophil count <1.0×10^9^/liter), death before evaluation by the investigators, injection drug use, surgery within 30 days, or indwelling prostheses. These exclusion criteria eliminate important non-pathogen characteristics that can influence the severity of staphylococcal disease.

Next, we used strict definitions to identify three phenotypically distinct clinical groups which represent a progression from healthy individuals to those who are severely affected: 1) nasal carriage, 2) uncomplicated infection, and 3) bacteremia with hematogenous complications. Nasal carriage isolates were obtained from the nares of non-infected subjects in two distinct epidemiologic settings within the DUMC referral area: a) healthy undergraduate students with no current infection or healthcare contact (community carriage isolates) and hospitalized or hemodialysis-dependent subjects with no active infection (healthcare-associated carriage isolates).

Isolates in the uncomplicated infection group were obtained from patients with uncomplicated bacteremia or soft tissue infection. Uncomplicated bacteremia isolates were obtained from the bloodstream of DUMC patients meeting all of the following criteria (in addition to the criteria listed above): a) confirmed intravascular catheter-associated *S. aureus* bacteremia [17], b) receipt of ≤14 days of antibiotics, c) no evidence of metastatic infection by both clinical evaluation and echocardiogram, and d) alive without complications 12 weeks after the initial blood culture. In addition, to ensure that study findings were not attributable to the single center design, MSSA isolates (n = 48) were obtained from skin and soft tissue infections of anonymous geographically diverse patients participating in a Phase II clinical trial (Vicuron Pharmaceuticals). These patients had a culture-confirmed soft-tissue *S. aureus* infection with signs of systemic illness, but sterile blood cultures documented at the time of enrollment.

Isolates from the third group, bacteremia with hematogenous complications, were from the bloodstream of patients with definite native aortic or mitral valve IE [18], hematogenous bone and joint infection, or both. Patients with bone and joint infection had *S. aureus* isolated from a vertebral body, intervertebral disk, or joint space. All patients with bacteremia were followed up 12 weeks after the initial positive blood culture to confirm that infection groupings were accurate.

### Microarray design

The *S. aureus* microarrays used for these experiments were obtained from the Pathogen Functional Genomics Resource Center (PFGRC) at The Institute for Genomic Research (TIGR, now the JCVI) (*S. aureus* version 3 microarrays). The arrays consist of 10,800 total spots, with 688 empty spots, 1,024 reserved spots containing Arabidopsis control oligonucleotides and 9,088 spots representing duplicate spotted oligos (n = 4544 70 base oligomers) of all known open reading frames in *S. aureus* at the time of array construction (N315 and Mu50 [18], MSSA476 and MRSA252 [19], COL [20] and MW2 [21]). The 688 empty spots were used as negative controls to measure the signal values that represent background in fluorescence.

### Determination of microarray probe specificity

Preliminary examination of hybridization probe specificity suggested that several microarray probes had the potential to hybridize to multiple targets. To evaluate the hybridization specificity of the array probes, we used BLAST to determine the similarity between all individual probe sequences and the open reading frames in the *S. aureus* genomes included on the array. BLAST was completed using a word size of 30 and the alignment results filtered to require 90% sequence overlap. The e-value and bit score were recorded for each probe. Since some probes matched multiple genes within one genome, the total number of hits with bit scores above 100 was also noted. All genes that had a significant probe hit were aligned using CLUSTAL and the resulting set was then collected and Hidden Markov Models [22] were used to predict function with the Interpro scan program. Probes representing known pathogenicity islands, virulence factors and surface proteins were manually identified and where possible, ordered to identify their relative position in the *S. aureus* genome.

### Identification of the *S. aureus* core genome and positive microarray controls

BLAST alignment of microarray probes (using criteria as described above) was used to identify which of the 4544 probes are hybridizing to genes shared among sequenced *S. aureus* genomes (COL, N315, MW2, Mu50, MRSA252 and MSSA476). Sequences with a bit score of greater than 100 in all sequenced genomes, excluding known virulence factors, surface proteins and genes in pathogenicity islands (PI) were designated as core (Table S1). The 1930 core probes represent 1660 genes. These probes were used as positive controls for aCGH analysis of the clinical strains. Of the remaining 2,614 probes, 600 were determined to not be variable in our sample based upon hybridization to all (or none) of the arrays (Table S1). Thus 2,014 variable probes remained, including virulence factors and other factors likely responsible for increased fitness or virulence. We used this definition of core and variable in case some of the known virulence factors were variable in our study population, even though they were not variable in the existing sequenced strains. We found this was the case and seven genes were included in our study in this way. These genes are serine proteases *splC* (SACOL1867), *splD* (SA1628) and *splF* (SA1627) and also *cap5M* (SACOL0148), cell wall hydrolase (SACOL1264), entertoxin homolog (SA1429) and hypothetical (SA0850).

### Selection of strains

A total of 379 isolates (254 MSSA and 125 MRSA) were in our initial study [9] based on narrow clinical groupings described above. The CGH analysis was designed to include all of the MRSA isolates (n = 125) and a 50% random sample of the 244 MSSA isolates with non-missing spa and MLST types, stratifying on clinical severity. Four MRSA isolates and 4 MSSA were excluded during quality control checks. Thus, the final aCGH sample for this study included 239 isolates (121 MRSA; 118 MSSA).

### Preparation of *S. aureus* genomic DNA

*S. aureus* isolates were grown overnight at 37°C in 1.5 ml Brain Heart Infusion (BHI) media. Genomic DNA was extracted from 500 µl of the cultures using the automated Qiagen M48 extractor and reagents (Qiagen, Valencia, CA), modified by the addition of lysostaphin (Sigma, St. Louis, MO) to the cell lysis buffer (final concentration of lysostaphin at 25 mg/ml).

### Array Comparative Genome Hybridization (aCGH)

For hybridization, genomic DNA (500 ng) was amplified in Klenow reaction buffer containing random oligonucleotide primers (Invitrogen, Carlsbad, CA), high activity Klenow (New England Biolabs, Ipswich, MA) and dNTP/ddUTP. Amplified DNA was coupled to cy3 or cy5 and purified on Qiagen columns (Qiagen, Valencia, CA) prior to hybridization. The arrays were hybridized, washed and scanned using standard methods [23]. A study on a set of already sequenced strains demonstrated that single replicates where calls of presence/absence were made relative to negative controls showed greater sensitivity and specificity than a reference design (data not shown). Accordingly, we developed a hybridization design where each sample was hybridized once. The groups (control (C), uncomplicated (U), and complicated/sick (S) were each balanced for dye such that half of the samples in a particular group were hybridized with cy3 and half of the samples with cy5 and different clinical groupings represented on each slide. The control group was considered the reference and 36 control strains were repeated. The strains to be repeated were chosen at random from among all control strains. Quality control on these repeated hybridizations found excellent agreement. Images were quantified with Imagene v.7. Local background signals were subtracted from the signal intensity of the spot. Imagene's automated spot flagging feature was used to identify and remove poor quality spots (*i.e.* those contaminated by small dust particles). Each slide was checked to ensure that less than 5% of the slide was affected by imperfections and poor quality. Spots were determined to be present if they were greater than 90% of the negative controls. Validation of the aCGH method is described in Supporting Information File S1.

### Associations

As the primary interest is identifying genes contributing to severe disease, the uncomplicated and control samples were combined into a single group, ‘not-complicated’, and compared to the group, ‘complicated infection’ for significance testing. The presence/absence of a particular probe was compared to this binary clinical outcome (not complicated vs. complicated) using a chi-squared test. P-values for this test were computed exactly as some comparisons had small cells [24]. A false discovery rate (FDR), of 0.20 was used to control for multiple testing [25], [26], [27]. This level was chosen to minimize the chance of failing to identify important loci. Further descriptive comparisons are reported to be significant at a nominal level of 0.05. All unadjusted p-values are reported in Table S2.

### PCR verification of microarray data

The presence/absence of the 14 genes associated with severity of illness was verified by PCR amplification of genomic DNA from the corresponding strains. The primers, which flank the 70-mer oligonucleotide probes for these genes, are listed in Table S3. PCRs were said to agree with array results if simple agreement was greater than 0.60.

## Results

We used aCGH to survey 239 clinical *S. aureus* isolates with the goal of identifying genes that may contribute to increased virulence. The genotypic characteristics of the study subjects with asymptomatic *S. aureus* nasal carriage, soft tissue infection, uncomplicated *S. aureus* bacteremia and complicated *S. aureus* bacteremia are listed in Table 1. Six CCs (CC 1, 5, 8, 15, 30, 45) out of 15 CCs total, were observed in 10 or more subjects in either the MRSA or MSSA subset in the current study. The *S. aureus* microarray used for this aCGH study allows for resolution of variations such as gene presence or absence as well as genomic rearrangements [12], but does not allow detection of individual DNA polymorphisms [11], [28]. Data from this study demonstrate: 1) genetic differences within the 239 isolates, including surface proteins and toxins, 2) genes unique to CC5 and CC30, 3) genes in the *mec* element associated with disease severity and 4) non-*mec* genes associated with disease severity (see Table S2).

**Table 1:** Genotypic characteristics of 239 study subjects.

	Sick^a^	Not Sick^b^
Characteristic	(N = 114)	(N = 125)
**Clonal Complex** c		
1	4/112 (3.6%)	14/125 (11.2%)
5	40/112 (35.7%)	26/125 (20.8%)
8	8/112 (7.1%)	11/125 (8.8%)
9	4/112 (3.6%)	0
15	2/112 (1.8%)	10/125 (8.0%)
30	39/112 (34.8%)	30/125 (24.0%)
45	8/112 (7.1%)	12/125 (9.6%)
59	1/112 (0.9%)	3/125 (2.4%)
Other^d^	6/112 (5.4%)	19/125 (15.2%)
**spa type** c		
1	3/111 (2.7%)	4/124 (3.2%)
2	29/111 (26.1%)	21/124 (16.9%)
16	24/111 (21.6%)	14/124 (11.3%)
17	1/111 (0.9%)	3/124 (2.4%)
33	4/111 (3.6%)	6/124 (4.8%)
139	0	2/124 (1.6%)
Other^e^	50/111 (45.0%)	74/124 (59.7%)

^(a)^Includes bacteremia with bone and joint infection only (n = 52), native valve endocarditis only (n = 55) and both bone and joint infection and native valve endocarditis (n = 7).

^(b)^Includes healthy subject nasal carriage (n = 31), healthcare-associated nasal carriage hemodialysis-dependent subjects (n = 20), (hospitilized subjects with no history of hemodialysis dependence (n = 18), soft tissue infection (n = 21) and uncomplicated bacteremia (n = 35).

^(c)^Clonal complex and spa types previously described (9) are reported here.

^(d)^12, 25, 72, 78, 97, 121, 395.

^(e)^3, 7, 12, 14, 15, 18, 21, 23, 29, 35, 37, 42, 43, 45, 47, 62, 65, 93, 97, 122, 131, 141, 151, 152, 155,163, 168, 175, 176, 184, 193, 194, 199, 203, 204, 220, 247, 314, 363, 390, 433, 437, 449, 466, 487, 493, 499, 501, 503, 504, 505, 506, 507, 509, 514, 517, 518, 520, 521, 522, 524, 527, 529, 530, 531, 535, 536, 537, 538, 540, 541, 544, 546, 547, 548, 549, 596, 597, 598, 599, 600, 601, 602.

### Frequency of gene presence or absence in clonal complex

Comparative genomic analysis revealed that the oligonucleotides included on the *S. aureus* microarray contain 1930 probes to ORFs common in all sequenced genomes at the time, and 2,614 variable probes. The variable component represents strain specific genes including virulence factors and surface proteins frequently present among the sequenced genomes. Analysis of our aCGH data found that 600 of these probes were not variable in our sample of 239 strains, with 569 always present and 31 always absent. The frequency among the 5 major CCs in our study was calculated for each of the remaining 2,014 probes that were variable in our population.

Within CC5 and CC30 previously associated with clinical outcome [9], there were 1232 (61%) sequences that differed in frequency, with 731 (38%) being more frequent in CC30 and 461 (23%) more frequent in CC5 (Table S2). We also compared the presence/absence of each probe sequence in CC5 with other (non CC30) clonal complexes and the presence/absence of each sequence in CC30 with other (non CC5) clonal complexes. We found 626 (31%) genes more frequently present in CC30 and 249 (12%) genes more frequently present in CC5. There were 98 genes more present in both CC5 and CC30 compared to other clonal complexes.

### Genes associated with the SCC*mec*-element (MRSA)

Our initial study demonstrated that MRSA status was associated with clinical outcome [9]. In order to understand the relationship between the 2,014 variable genes and the SCC*mec* element and MRSA status, we compared the presence/absence of genes in MRSA and MSSA strains. We found 140 genes significantly more frequent in MRSA and 1079 genes more frequent in MSSA (Table S2).

### Genes associated with severe infection

**Figure 1:**
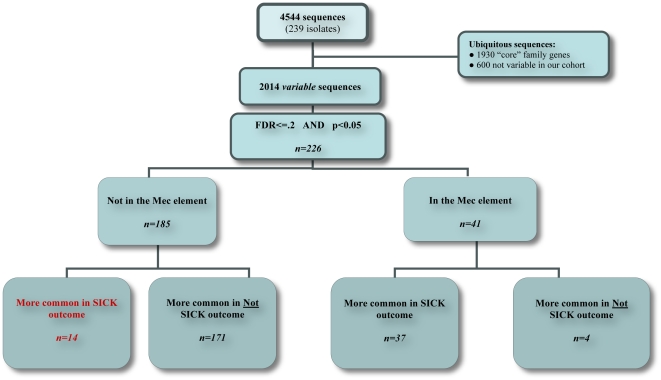
Disposition of 4544 aCGH probes. This figure illustrates how we arrived at our candidate genes of interest. Of the 2014 sequences and 239 isolates, a total of 226 sequences were statistically significant after multiple comparisons adjustment. Of these 226 sequences, 185 were not and 41 were in the SCC*mec* element. Most of the 185 non-SCC*mec* genes-171-were significantly less common in the complicated infection group. The remaining 14 genes were significantly more common in isolates from the complicated infection group. Among the 41 SCC*mec* element genes, 37 were significantly more common in the complicated group and 4 were significantly less common in the complicated group. Therefore, a total of 51 of the 2014 sequences, 14 non-mec associated and 37 mec associated, were identified as genes of interest.

**Figure 2:**
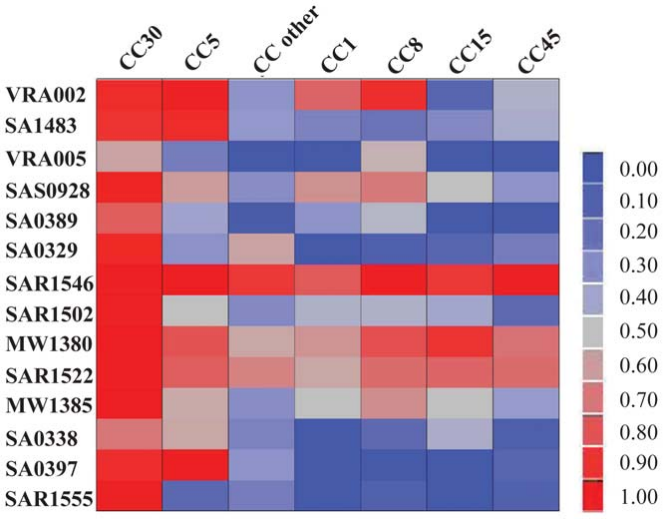
Distribution of 14 genes associated with complicated infections across clonal complexes. Proportion of isolates that have the 14 genes in each clonal complex shown on a color gradient with 1 being bright red and 0 being bright blue. All 14 genes are more abundant in CC5 and 30, the clonal complexes previously shown to exhibit a significant trend toward increasing levels of hematogenous complications.

From the 2014 variable sequences, the presence/absence of 226 sequences were statistically significantly associated with severity of infection (Figure 1 and Table S3). Within these 226 sequences, there were 41 genes known to be part of a SCC*mec* element. Among these 41 genes, 37 were present more frequently in the complicated group. Of the 185 genes not in the SCC*mec* element, 14 were present more frequently in the complicated group. For this study we further focused on these 14 non-SCC*mec* associated genes which were more common in the complicated group (Table 2). The relative proportion of these 14 genes in each of the major clonal complexes in our study is shown in Figure 2.

**Table 2:** Putative virulence genes associated with complicated infections.

				Clinical Outcome			
Common Gene Name	Locus	Source Strain	Sum Hits^a^	Array P-value For Association^b^	PCR P-value For Association^c^	P-value After Adjust for MRSA^d^	Simple Agreement
transposase/integrase	VRA0002	Michigan-VRSA	13	0.0008	0.0007	0.5604	0.927*
transposase/integrase	SA1483	N315	3	0.0004	<0.0001	0.0332	0.899*
thymidylate synthase	VRA0005	Michigan-VRSA	1	0.0147	0.152	0.6199	0.89*
hypothetical phage protein	SAS0928	MSSA476	1	0.0127	0.0018	0.2105	0.99*
prophage amidase/cell wall autolysin	SA0389	COL	2	0.0112	0.0019	0.4321	0.867*
conserved hypothetical protein	SA0329	COL	1	0.0205	0.1916	0.4568	0.838*
hypothetical phage protein	SAR1546	MRSA252	2	0.0227	0.0001	0.0261	0.581
hypothetical phage protein	SAR1502	MRSA252	1	0.0131	0.0019	0.1661	0.768*
prophage amidase/cell wall autolysin	MW1380	MW2	2	0.0124	0.0029	0.2461	0.853*
phage regulatory protein	SAR1522	MRSA252	4	0.002	n.d.	n.d.	n.d.
hypothetical protein	MW1385	MW2	2	0.0022	0.0011	0.1764	0.994*
conserved hypothetical protein	SA0338	COL	1	0.0085	<0.0001	0.0132	0.988*
hypothetical protein	SA0397	N315	3	0.0049	0.0219	0.4199	0.755*
putative regulatory protein	SAR1555	MRSA252	1	0.0146	0.0079	0.7332	0.908*

^(a)^Sum Hits: the number of times this probe hybridized to a part of the sequenced genomes.

^(b)^P-value for clinical association from aCGH results.

^(c)^P-value for clinical association from PCR validation.

^(d)^P-value for gene association with sick outcome after adjusting for MRSA status.

*Value greater than 0.60 indicates PCR results are in simple aggrement with a CGH results.

### Verification of microarray findings

The results of the aCGH analysis were verified by PCR amplification for 13 of the 14 loci described above. Amplification of PCR primers failed for one gene (SAR1522). For 12 out of the 13 genes (92.3%), the concordance between the array presence/absence data and the PCR validation was excellent. In comparison, the concordance of most large-scale array vs. PCR comparisons [29], [30] is ∼60%, clearly demonstrating the quality of our aCGH data. For genes that were PCR validated, the association with severity of infection is reported based upon the PCR results rather than the array results (Table 2).

## Discussion

The ability of *S. aureus* to cause severe infections is dependent upon a large repertoire of virulence factors, often on mobile genetic elements or MGE (including pathogenicity islands and bacteriophage) that are transferred horizontally through the *S. aureus* community. The virulence capacity of individual isolates is largely determined by the function and abundance of these variable virulence factors. In our previous work [9], we demonstrated that *S. aureus* CC5 and CC30 are associated with the presence of hematogenous complications, including endocarditis, septic arthritis and vertebral osteomyelitis. We now undertake aCGH analysis to examine the association of 2014 variable *S. aureus* genes with severity of infection in a collection of 239 isolates with the goal of identifying genes that contribute to virulence. The 239 *S. aureus* isolates were selected from three clinical groups in our previous study [9] and included 121 MRSA and 118 MSSA isolates. Of the 226 genes that were identified as significantly associated with severity of infection, 51 were more frequent and 175 less frequent in the complicated infection group (Table S4).

We acknowledge that as a result of the microarray probe design being limited to the six genomes available when the microarray was constructed, our analysis is compromised and will not capture those genes present in many other *S. aureus* lineages and strains. As examples, genes encoding numerous heavy metal resistance genes on the SCC*mec* type III element as well as genes encoding the *lukSF-PV* Panton Valentine Leukotoxin and ACME element from Community Acquired MRSA (CA-MRSA) will not be identified in our analysis. However, a significant strength of our study is identification of the core *S. aureus* ORFs present in all tested clinical strains representing fifteen clonal complexes.

To minimize SCC*mec* as a confounding factor, the 226 variable genes were segregated into two groups, based on their presence in the SCC*mec* element (Figure 1). Of the 51 genes more common in complicated infection, 37 were carried on SCC*mec* and 14 were not carried on SCC*mec*. None of these 14 genes encode known virulence factors previously associated with increased virulence and disease severity. Instead, 11 genes include proteins of unknown function, regulatory proteins and autolysins carried on *S. aureus* bacteriophage. These findings support previous work demonstrating the contribution of *S. aureus* bacteriophage to pathogenesis of infections [31], [32], [33], [34] through genome variation, mobilization of pathogenicity islands and transmission of phage encoded virulence factors.

Yet to be resolved is the contribution of prophage to potentiate *S. aureus* virulence by influencing its physiology towards growth as a biofilm, a virulence factor that plays a role in chronic infections, such as native valve endocarditis and osteomyelitis. Evidence of bacteriophage release from *S. aureus* biofilms has been reported [35], leading to lysis of cells which in turn promotes the persistence and survival of remaining cells and release of extracellular DNA (eDNA) required for extracellular polymeric substance (EPS) development and maturation of biofilms. A second mechanism for cell lysis and release of eDNA required for biofilm development involves autolysis driven by autolysins and regulated by the *cid* and *lrg* loci [36]. These autolysins are capable of hydrolyzing the amide bond between N-acetylmuramic acid and L-alanine in bacterial cell-wall peptidoglycan resulting in cell lysis and release of eDNA. Interestingly, two of the bacteriophage genes more frequently present in complicated infections are autolysins carried on bacteriophages Sa2MW (MW1380) and L54a (SACOL0389) (Table 2). Although, no role has yet to be assigned to these two putative autolysin genes, their presence in cases of complicated infection suggests that they may influence biofilm maturation and disease persistence. An alternative function for the *S. aureus* phage encoded autolysins is suggested by recent work on bacteriophage-encoded autolysins in *Streptococcus mitis*, which demonstrated their role in binding to human platelets through interaction with fibrinogen [37].

Of the remaining 9 genes carried on prophage, three are DNA binding proteins with potential regulatory functions (SAS0928, SAR1502 and SAR1555) and six (SACOL0329, SAR1546, SAR1522, MW1385, SACOL0338 and SA0397) are genes of unknown function. The function of three remaining genes (VRA0002, SA1483 and VRA0005) relative to virulence is also not clear. Association of these genes with increased disease severity may be a result of their linkage to unidentified genes not on the microarray and associated with virulence. This study did not start with candidate genes, and as such uncovered a list of genes for which little is known about the function. Detailed functional analysis of these and other *S. aureus* bacteriophage genes of unknown function will likely lead to a greater understanding of their role in virulence and enabling mutation and adaptation of *S. aureus* to new environments.

The 37 genes that were more frequently present in complicated infection and carried by SCC*mec* span the entire length of the type II SCC*mec* element, which is found in the majority (97/120; 81%) of MRSA isolates included in this study. While there is no direct evidence that SCC*mec* contributes to *S. aureus* virulence, recent work by Queck *et. al.* [38] has shown the type II SCC*mec* elements encode a new phenol soluble modulin (PSM) that alters the capacity of *S. aureus* to cause disease. Although the arrays used in this study do not contain a probe for this PSM, two genes flanking the PSM encoding region in SCC*mec* type II, *mecI* (N315-SA0039) and *xylR* (N315-SA0041) are associated with severity of infection, suggesting that the new PSM may also be a significant component in our study. However, sequence analysis of this region showed that the PSM is present in all members of a subset of 69 isolates from the study group representing all 3 clinical groups and student controls. Outside of the SCC*mec* element, there were 140 genes associated with SCC*mec* and severity of infection (Table S4). This is intriguing as it suggests the possibility of co-evolution of genes in the *S. aureus* genome with SCC*mec* and the possible preferential insertion of SCC*mec* into specific strains.

An unexpected finding in our study was the identification of 171 genes more frequent in strains isolated in subjects with uncomplicated infection. These genes (included in Table S4) include 152 genes of unknown function, some of which may be involved in attenuation of virulence. Approximately 38% of *S. aureus* genes have no assigned function and an additional 10% have a putative function based on functional domain matches [39]. Their roles as genes that contribute to *S. aureus* specific functions such as pathogenicity or host niche adaptation, remains an unexplored area of this significant bacterial pathogen.

In summary, we have demonstrated a statistical association between the presence/absence of individual genes and clinical outcome. Our data strongly supports the concept that differences in disease potential are rooted in the *S. aureus* genotype. However, the use of aCGH in our current study limits the results to identification of large-scale genomic changes, such as gain or loss of genes, responsible for phenotypic changes. Additional genotypic factors, including genomic polymorphisms, novel genes and differential gene expression, associated with complicated infections, will be determined by resequencing of representative members of the SABG collection and global transcriptional analysis from *in vitro* and *in vivo* environments. Correlation of these genomic and transcriptional differences with clinical outcome will contribute significantly to our understanding of *S. aureus* virulence.

## Supporting Information

File S1 Supplementary Text and Description of Tables S1, S2 and S3.
(DOC)

Table S1 Ubiquitous genes described in Figure 1 (1930 core family genes and 600 not variable in our cohort).
(XLS)

Table S2 Overall association of *S. aureus* genes with clinical outcome.
(XLS)

Table S3 Primers used in this study.
(XLS)

Table S4 Genes statistically significantly associated with severity of infection.
(XLS)
