# Supplementary material for: Potential Associations between Severity of Infection and the Presence of Virulence-Associated Genes in Clinical Strains of Staphylococcus aureus
Source: PLoS One. 2011 Apr 26;6(4):e18673. doi: 10.1371/journal.pone.0018673 (PMC3082525; doi:10.1371/journal.pone.0018673)
Supplement: File S1 — Supplementary Text and Description of Tables S1, S2 and S3. (DOC) [file pone.0018673.s001.doc]

**Supporting Information**

**Validation of aCGH method.** The range of hybridization intensity values from the positive controls (core genome ORFs) and negative controls (empty spots on the microarray) were used to determine ORF presence and absence. Genes that are present fall within the distribution of positive controls and genes that are absent fall within the distribution of negative controls. To clearly separate the distribution of positive and negative controls, we used the 5% value of the positive controls and the 95% value of the negative controls. Signal values falling in between these two levels are considered ambiguous. In our analysis of data obtained from aCGH analysis with sequenced *S. aureus* genomes (unpublished data), we have shown that this approach has less than a 3% false negative rate and less than a 1% false positive rate. In addition, the agreement for the same sample across dyes was very high (kappa > .9), therefore eliminating the need for technical replicates. In the rare cases where the two replicates disagreed in presence/absence, the ORF was deemed ambiguous in further analysis. Three slides in which the positive and negative control distributions overlapped were considered poor quality hybridizations and eliminated from further analysis, leaving 121 subjects for analysis in the MRSA group and 118 in the MSSA group. As a final check on slide quality, hybridizations of seven strains were randomly repeated and compared for presence/absence for all variable ORFs.

**Documentation of Table S1: Ubiquitous genes described in Figure 1 (1930 core family genes and 600 not variable in our cohort).**

**Name in Table Description**

**sequence**  the sequence analyzed

**common_name**  Common name

**locus**  Locus ID

**strain**  Strain

**core**  Genes that are common to all genomes

on the microarray

1=core family gene

0=not a core family gene

**always_present** For the 600 not variable sequences, indicates

whether genes is always present or

always absent

1=sequence always present

0=sequence always absent

blank=sequence not examined in our study

**Documentation of Table S2: Overall association of *S. aureus* genes with clinical outcome.**

**Name in Table Description**

**sequence**  the sequence analyzed

**common_name**  Common name

**locus**  Locus ID

**strain**  Strain

**pi**  Pathogenicity Island indicator

**vf**  Virulence factor indicator

**sp**  Surface protein indicator

**mec_indicator**  Indicator for sequence in mec element

1=sequence in mec element

0=sequence not in mec element

**percent_ambiguous** Percent ambiguous calls

**p_association**  Nominal p value from overall test of gene association with sick outcome

**fdr_association**  FDR p value from overall test of gene association with sick outcome

**significant_association** Indicator for 226 significant genes

(FDR<=.2 & p_association<.05 in overall

test of gene association with sick outcome)

**selected_for_verification** Indicator for 14 significant genes

(FDR<=.2 & p_association<.05 &

mec_flag=0 & more present in sick outcome)

**p_mrsa_vs_mssa**  Nominal p value from overall test of gene association with MR vs MS

**prop_pres_sick**  Proportion present in sick

**prop_pres_notsick** Proportion present in the Not Sick

**prop_pres_MRSA** Proportion present in MRSA

**prop_pres_MSSA** Proportion present in MSSA

**prop_pres_CC1** Proportion present in CC 1

**prop_pres_CC5**  Proportion present in CC 5

**prop_pres_CC8**  Proportion present in CC 8

**prop_pres_CC15**  Proportion present in CC 15

**prop_pres_CC30**  Proportion present in CC 30

**prop_pres_CC45** Proportion present in CC 45

**prop_pres_other**  Proportion present in other CC’s

(not 1, 5, 8, 15, 30, 45)

**Documentation of Table S4: Genes statistically significantly associated with severity of infection.**

**Name in Table Description**

**sequence**  the sequence analyzed

**common_name**  Common name

**locus**  Locus ID

**strain**  Strain

**pi**  Pathogenicity Island indicator

**genetic element** Element indicator

**vf**  Virulence factor indicator

**sp**  Surface protein indicator

**mec_indicator**  Indicator for sequence in mec element

1=sequence in mec element

0=sequence not in mec element

**percent_ambiguous** Percent ambiguous calls

**p_association**  Nominal p value from overall test of gene association with sick outcome

**fdr_association**  FDR p value from overall test of gene association with sick outcome

**sccmec_more_common_sick 37 genes**

**sccmec_more_common_notsick 4 genes**

**not_sccmec_more_common_sick 14 genes**

**not_sccmec_more_common_notsick 171 genes**

**selected_for_verification** Indicator for 14 significant genes

(FDR<=.2 & p_association<.05 & mec_flag=0 & more present in sick outcome)
